# Preclinical Evaluation of 1,2-Diamino-4,5-Dibromobenzene in Genetically Engineered Mouse Models of Pancreatic Cancer

**DOI:** 10.3390/cells8060563

**Published:** 2019-06-09

**Authors:** Robert G. Goetze, Soeren M. Buchholz, Ning Ou, Qinrong Zhang, Shilpa Patil, Markus Schirmer, Shiv K. Singh, Volker Ellenrieder, Elisabeth Hessmann, Qing-Bin Lu, Albrecht Neesse

**Affiliations:** 1 Department of Gastroenterology and Gastrointestinal Oncology, University Medicine Goettingen, 37075 Goettingen, Germany; robert.goetze@med.uni-goettingen.de (R.G.G.); soeren.buchholz@stud.uni-goettingen.de (S.M.B.); shilpa.patil@med.uni-goettingen.de (S.P.); shiv.singh@med.uni-goettingen.de (S.K.S.); volker.ellenrieder@med.uni-goettingen.de (V.E.); elisabeth.hessmann@med.uni-goettingen.de (E.H.); 2 Department of Physics and Astronomy, University of Waterloo, Waterloo, ON N2L 3G1, Canada; nou@uwaterloo.ca (N.O.); qinrong.zhang@berkeley.edu (Q.Z.); qblu@uwaterloo.ca (Q.-B.L.); 3 Department of Radiotherapy and Radiation Oncology, University Medicine Goettingen, 37075 Goettingen, Germany; mschirmer@med.uni-goettingen.de

**Keywords:** 1,2-Diamino-4,5-dibromobenzene, femtomedicine compounds, pancreatic cancer, GEMMs, chemoresistance, radiation therapy, radiosensitizer

## Abstract

**Background:** Pancreatic ductal adenocarcinoma (PDAC) is highly resistant to standard chemo- and radiotherapy. Recently, a new class of non-platinum-based halogenated molecules (called FMD compounds) was discovered that selectively kills cancer cells. Here, we investigate the potential of 1,2-Diamino-4,5-dibromobenzene (2Br-DAB) in combination with standard chemotherapy and radiotherapy in murine and human PDAC. **Methods:** Cell viability and colony formation was performed in human (Panc1, BxPC3, PaTu8988t, MiaPaCa) and three murine *LSL-Kras^G12D/+^;LSL-Trp53^R172H/+^;Pdx-1-Cre* (KPC) pancreatic cancer cell lines. In vivo, preclinical experiments were conducted in *LSL-Kras^G12D/+^;p48-Cre* (KC) and KPC mice using 2Br-DAB (7 mg/kg, i.p.), +/- radiation (10 × 1.8 Gy), gemcitabine (100 mg/kg, i.p.), or a combination. Tumor growth and therapeutic response were assessed by high-resolution ultrasound and immunohistochemistry. **Results:** 2Br-DAB significantly reduced cell viability in human and murine pancreatic cancer cell lines in a dose-dependent manner. In particular, colony formation in human Panc1 cells was significantly decreased upon 25 µM 2Br-DAB + radiation treatment compared with vehicle control (*p* = 0.03). In vivo, 2Br-DAB reduced tumor frequency in KC mice. In the KPC model, 2Br-DAB or gemcitabine monotherapy had comparable therapeutic effects. Furthermore, the combination of gemcitabine and 2Br-DAB or 2Br-DAB and 18 Gy irradiation showed additional antineoplastic effects. **Conclusions:** 2Br-DAB is effective in killing pancreatic cancer cells in vitro. 2Br-DAB was not toxic in vivo, and additional antineoplastic effects were observed in combination with irradiation.

## 1. Introduction

Pancreatic ductal adenocarcinoma (PDAC) is one of the most aggressive malignancies worldwide highlighted by a balanced incidence and mortality rate [1]. Despite intense scientific and clinical efforts, the five-year survival rate is below 8%. Only 20% of all newly diagnosed patients are eligible for curative treatment achieved by a combination of surgical resection and adjuvant chemotherapy with gemcitabine monotherapy [2], or gemcitabine + capecitabine [3]. Recent data suggest that adjuvant administration of a combination chemotherapy with fluorouracil, leucovorin, irinotecan, and oxaliplatin (FOLFIRINOX) may yield a significant survival benefit of 54 months versus 35 months for gemcitabine monotherapy [4]. However, the majority of patients are diagnosed with advanced or metastatic disease and receive palliative chemotherapy. To this end, FOLFIRINOX, nab-paclitaxel + gemcitabine or gemcitabine monotherapy can be given dependent on general morbidity and performance status [5,6]. However, antineoplastic agents, in particular intensified chemotherapies such as FOLFIRINOX, cause severe side effects such as neutropenia, sepsis, diarrhea, and nausea [7]. Therefore, novel, potentially more cancer-specific compounds are urgently needed for effective therapy of PDAC.

Cisplatin is a widely used chemotherapy drug for several types of cancer and mediates cytotoxicity through direct binding of the cis-[Pt(NH_3_)_2_] unit to DNA that stalls cell proliferation and activates DNA damage response [8,9]. In clinical routine, cisplatin can be used as a single agent or in combination with ionizing radiation [10]. In this regard, high energy radiation in biological systems induces radiolysis of water, an unbalanced chemical reaction leading to the generation of hydroxyl radicals (OH**^∙^**) and free electrons with high kinetic energies. As electrons are rapidly hydrated (e^−^_hyd_), the OH**^∙^** radical was thought to mainly cause DNA damage. Using femtosecond time-resolved laser spectroscopy, a prehydrated state of electrons (e^−^_pre_) was observed that lasts less than a picosecond before the full hydration occurs [11]. Despite the fact that e^−^_pre_ is ultrashort-lived and weekly-bound, its reactions with nucleotides form transient anions such as dGMP^−^ and dTMP^−^ that dissociate to cause chemical bond breaks in them and then single- and double-strand breaks in DNA, a mechanism termed dissociative electron transfer (DET) [12,13]. Interestingly, DET was also observed for cisplatin where its DET reaction with the ultrashort-lived e^−^_pre_ or the G base in DNA forms the *cis*-Pt(NH_3_)_2_Cl^•^ or *cis*-Pt(NH_3_)_2_^•^ radical to induce DNA damage either in combination with radiotherapy or as chemotherapy alone [14,15]. Weakly bound electrons have been thought to occur more frequently in a reductive environment in cancer cells with a high concentration of antioxidants [16,17], a fact that may open a window for a “targeted” chemotherapy with reduced side effects on normal proliferating cells. Indeed, cisplatin regularly causes pronounced neuro- and nephrotoxicity that has been mainly associated with the heavy-metal platinum.

Recently, based on the DET mechanism and the femtomedicine (FMD) strategy [18], a new class of non-platinum-based halogenated compounds (called “FMD compounds”) was discovered that features an aromatic ring coupled to two NH_2_ groups serving as an electron transfer promoter, and at least one halogen atom as a leaving group [19]. FMDs are structurally closely related to cisplatin, but lack the platinum ion that causes the majority of toxic side effects. Notably, a DET reaction is also found in FMDs and may contribute to the cytotoxic potential of FMDs as single chemotherapeutic agents [19], or in combination with radiation therapy [20]. Indeed, the first preclinical studies using cell lines and xenograft models of cervical, lung, breast, head/neck-, and ovarian cancer revealed that FMDs caused minimal toxicity towards normal cells/tissues while sufficiently killing cancer cells [19,20]. However, FMDs have not been tested for PDAC that exhibits a high degree of chemoresistance and a pronounced desmoplastic reaction. Furthermore, well established and validated genetically engineered mouse models (GEMMs) are available for PDAC that might be more predictive for the response to novel therapies [21,22,23,24,25]. To this end, the *LSL-Kras^G12D/+^;p48-Cre* (KC) mouse model harbors an activating oncogenic Kras^G12D^ mutation that is conditionally activated via the pancreas-specific expression of Cre recombinase using the p48Cre promoter. KC mice develop endogenous precursor lesions such as acinar to ductal neoplasia (ADMs) and pancreatic intraepithelial neoplasia (PanINs) over the course of several months, and partially progress to frank adenocarcinomas of the pancreas after a latency of usually 8–15 months [26]. Therefore, the KC mouse model is ideally suited to investigate biologic aspects of pancreatic tumor progression, or therapeutic strategies that may prevent or slow down PanIN and carcinoma progression [27]. In addition, the *LSL-Kras^G12D/+^;LSL-Trp53^R172H/+^;Pdx-1-Cre* (KPC) mice have been generated to accelerate murine PDAC development by additionally introducing an inactivating mutation of the tumor suppressor gene p53 [28]. KPC mice develop invasive and metastatic PDAC within 3–6 months and are ideally suited to conduct preclinical therapeutic testing of new investigational compounds [29]. Importantly, both GEMMs closely recapitulate human histology featuring a pronounced desmoplastic reaction around preneoplastic and neoplastic cells, rendering them ideal model systems to test therapeutic resistance in this context.

Therefore, here we investigate the preclinical efficacy of the FMD compound 1,2-Diamino-4,5-dibromobenzene (2Br-DAB) in murine and human pancreatic cancer cell lines and both the KC and the KPC mouse models. 

## 2. Materials and Methods

### 2.1. In Vivo Studies Using Genetically Engineered Mouse Models 

The toxicity of 2Br-DAB was determined in a pilot study using KC mice (*n* = 4). 2Br-DAB (7 mg/kg) [19,20] was injected by intraperitoneal injection three times a week over 30 days. Survival/prevention studies were performed using the KC model. KC mice develop preneoplastic lesions like ADMs and PanINs within the first months of age, but only partially progress to PDAC within 8 to 15 months. Five-month-old KC mice (*n* = 31) were intraperitoneally injected with either 2Br-DAB (7 mg/kg; *n* = 16) or vehicle (10% ethanol, *n* = 15) once per week for a time course of 12 weeks. Mice were manually palpated for tumor formation on a weekly basis. For survival analysis, the endpoint criteria were defined as 20% body weight loss, general morbidity, lethargy, lack of physical activity, or development of ascites as previously described [30]. Survival days were counted from the initiation of therapy.

### 2.2. Therapeutic Intervention Study

KPC mice were used for the therapeutic intervention study. Before study enrollment, detection and evaluation of tumor-bearing mice were performed via weekly manual palpation and high- resolution ultrasound. Mice with confirmed PDAC and tumor diameters of 5–9 mm were enrolled in four different therapeutic treatment arms: gemcitabine (100 mg/kg), 2Br-DAB (7 mg/kg), gemcitabine/2Br-DAB, or vehicle. KPC mice were intraperitoneally injected four times with gemcitabine, 2Br-DAB, or vehicle over 11 days. To determine tumor growth, a follow-up ultrasound was performed during treatment days 7 and 10. Tumor diameters in two different planes were acquired and the volume of the corresponding PDAC was calculated using the formula of an ellipsoid [29]. 

All mice were housed at the local animal facility of the University Medical Center Goettingen in individually ventilated cages at a 12 h light, 12 h dark cycle. Prior to treatment, mice were separated and closely checked for health conditions. All animal studies were conducted in accordance with the national animal welfare regulations and after permission by the local authority (number: 33.9-42502-04-15/2056).

### 2.3. High-Resolution-Ultrasound

Starting at eight weeks of age, KPC mice underwent weekly abdominal palpations. Once a tumor suspicious abdominal mass was detected, animals were transferred to high-resolution ultrasound using the Visual Sonics Vevo 2100 System (FUJIFILM VisualSonics, Toronto, Canada) with microscan transducer MS-550-D, 22-55MHz (FUJIFILM VisualSonics, Toronto, Canada). The scanning protocol and procedures have been previously described by our group in detail [29].

### 2.4. Irradiation of Tumor-Bearing KPC Mice

Prior to radiation, the location of pancreatic tumors (head, body, or tail of pancreas) was determined by high-resolution ultrasound and marked on the abdominal wall. Therapeutic irradiation was delivered with a tube voltage of 200 kV and 15 mA (Siemens XStrahl RS225, Erlangen Germany) as previously described [31]. For a reliable and targeted irradiation of murine pancreatic tumors, all animals were constantly kept under sedation achieved by inhalation anaesthesia. The radiation device was equipped with a specialized working platform with a fixation stage including removable and adjustable lead plates (thickness of 25 mm) to reduce irradiation of the large and small bowel, anaesthesia induction chamber including isoflurane vaporizer, adjustable anaesthetic tubes, and a space holder. Because of the unavailability of a rotatable rack for continuous arc irradiation, irradiation was achieved through a single anterior to posterior beam at a dose of 1.8 Gy per session (2.5 Gy/min), conducted 10 times over the treatment course of 12 days. To test 2Br-DAB + radiation, mice were irradiated 1 h after the intraperitoneal injection of 2Br-DAB, according to a previously established protocol [19,20].

### 2.5. Drugs

1,2-Diamino-4,5-dibromobenzene (2Br-DAB) was obtained from Lu’s laboratory and administered at 7 mg/kg bodyweight [19,20]. Because of the precipitation of 2Br-DAB in hydrophilic liquids, a stock solution was prepared by resuspending 2Br-DAB in 100% ethanol at a final concentration of 10 mg/mL. Prior to intraperitoneal injection, the 2Br-DAB stock solution was further diluted to a final ethanol concentration of 10%. Gemcitabine hydrochloride (Sigma, Taufkirchen, Germany) was resuspended in sterile normal saline at 10 mg/mL. For all in vivo experiments, the final gemcitabine concentration was set to 100 mg/kg bodyweight. As the vehicle control, 10% ethanol dissolved in sterile saline was taken. 

### 2.6. Cell Lines

The human pancreatic cancer cell lines Panc1, BxPC3, MiaPaCa, and PaTu8988t were employed for in vitro experiments. In addition, three murine cell lines were derived from KPC tumors and maintained in DMEM (Thermo Fisher, Waltham, MA, USA) +10% FBS (Thermo Fisher, Waltham, MA, USA) as previously described [21,32]. Murine pancreatic stellate cells (mPSCs) were isolated from B6 mice and immortalized using SV40 large T antigen transfection as previously described [32,33].

### 2.7. In Vitro Cell Viability Assays

Pancreatic cancer cell lines were seeded with 5000 cells/well on a 96-well plate and were allowed to attach for 24 h. After medium change, cells were treated with either gemcitabine or 2Br-DAB and vehicle. Furthermore, cell lines were irradiated with 2, 4, 6, or 8 Gy after 2Br-DAB pre-incubation for 1 h. 72 h after treatment, MTT reagent (thiazolyl blue tetrazolium bromide, Sigma, Taufkirchen, Germany) was added to the media with a final concentration of 0.5 mg/mL and incubated for 2 h at 37 °C. Absorption was measured at 595 nm (PHOmo Microplate reader, Autobio Labtec Instruments, Zhengzhou City, China). Cell viability was expressed relative to controls. 

### 2.8. In Vitro Clonogenic Assay 

For all clonogenic assays, the seed-after-treatment approach was used [34]. Prior to treatment, cells were seeded in six-well plates with 30,000 cells/µL in a total volume of 2 mL and were allowed to attach for 24 h. Subsequently, 2Br-DAB was administered at a final concentration of 25 µM and pre-incubated for another 24 h before cells were subjected to irradiation with 2 Gy, or increasing doses of 2, 4, 6, and 8 Gy. Following irradiation, cells were washed twice in PBS, resuspended in fresh medium and plated in triplicates on a 100mm dish at a density of 3000 cells/dish, and incubated for 14 days at 37 °C. Fixation was achieved by using crystal violet staining. Colonies were counted manually by microscopy.

### 2.9. Immunohistochemistry 

Mouse tissues were fixed in 10% neutral buffered formalin (Sigma, Taufkirchen, Germany) for 24 h, dehydrated, and embedded in paraffin blocks. Then, 3–5 μm sections were processed for haematoxylin and eosin staining and immunohistochemistry using standard protocols as previously described [35]. Images were acquired with 40× magnification on an Olympus DP27 microscope using the Olympus cellSens Entry 1.12 software (Olympus, Hamburg, Germany). All acquired data were manually quantified by counting positive stained cells for Ki67 and γH2AX for at least seven high power fields (HPFs) divided by the number of all nuclei as determined by ImageJ. The following antibodies and kits were used: Ki67 (Thermo Scientific, Waltham, MA, USA, RM-9106-so, 1:400 dilution); γH2AX (Cell signalling, #9718, 1:200 dilution).

### 2.10. Statistical Analysis

Graph Pad Prism (version 7.03, GraphPad Software, San Diego, CA, USA) was used for statistical analysis. Data are shown as mean ± SD or box-plots with min–max values. Statistical significance was considered for *p* < 0.05. The Mann–Whitney U test was used for analysis, if not stated otherwise. 

## 3. Results

### 3.1. 2Br-DAB Is an Effective Cytotoxic Drug in Pancreatic Cancer Cell Lines 

**Figure 1:**
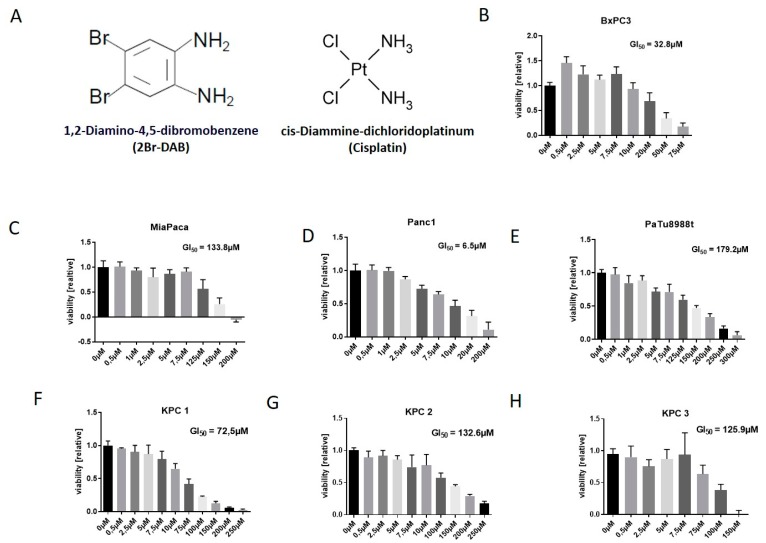
(**A**): Chemical structure of 2Br-DAB and cisplatin showing an aromatic ring system (2Br-DAB) or a central platin ion (cisplatin). Both molecules coordinate two halogenids and two amino residues [19]. (**B**–**H**): In vitro cell viability assays conducted in four different human ((**B**–**E**): Panc1, BxPC3, MiaPaca, PaTu8988t) and three murine (**F**–**H**) pancreatic cancer cell lines. After 72 h treatment with 2Br-DAB in various concentrations, cell viability was obtained by thiazolyl blue tetrazolium bromide (MTT) assay. Each graph represents results of at least two independent experiments. KPC—*LSL-Kras^G12D/+^;LSL-Trp53^R172H/+^;Pdx-1-Cre*.

1,2-Diamino-4,5-dibromobenzene (2Br-DAB) features an aromatic ring coupled to two NH_2_ groups and two halogen atoms. Importantly, 2Br-DAB is structurally closely related to cisplatin, but lacks the platinum ion that is associated with the majority of cisplatin induced side-effects (Figure 1A) [19]. 2Br-DAB was used for in vitro experiments on several well established murine and human pancreatic cancer cell lines. The human pancreatic cancer cell lines Panc1, Miapaca, BxPC3, and PaTu8988t cell showed GI_50_-values between 6 µM and 180 µM, with Panc1 being the most sensitive cell line and PaTu8988t the most resistant (Figure 1B–E). In addition, three different murine KPC cell lines were incubated with increasing concentrations of 2Br-DAB for 72 h, and cell viability was measured by MTT assay. All three murine KPC cell lines showed a dose-dependent reduction of viability with GI_50_ values ranging between 72 and 132 µM (Figure 1F–H). The most prominent effect was observed in KPC-1 (Figure 1F). These results show for the first time that 2Br-DAB is able to reduce cell viability in murine and human PDAC cell lines, consistent with the in vitro results of FMDs in treating the cell lines of other cancers [19].

### 3.2. FMD 2Br-DAB Is Well Tolerated In Vivo and Slows Tumor Progression in the KC Model 

Prior to testing 2Br-DAB in relevant GEMMs of PDAC, we performed a pilot study in KC mice (*n* = 4) to test the general toxicity of the compound in GEMMs. To this end, we administered 2Br-DAB at 7mg/kg three times a week over the course of 30 days and compared the effects to vehicle treated KC mice (10% ethanol). As side effects of cisplatin treatment in mice are well documented [19,36], we omitted a cisplatin control group based on the 3R principle for humane animal experiments. Clinical observation, body weight curves, and histological findings in highly proliferative tissues (small bowel) did not indicate any severe impact on the health of mice over the course of 30 days (Figure S1A,B). These results are consistent with the general toxicity of the FMD observed in non-genetically-engineered mouse models [19,20]. 

**Figure 2:**
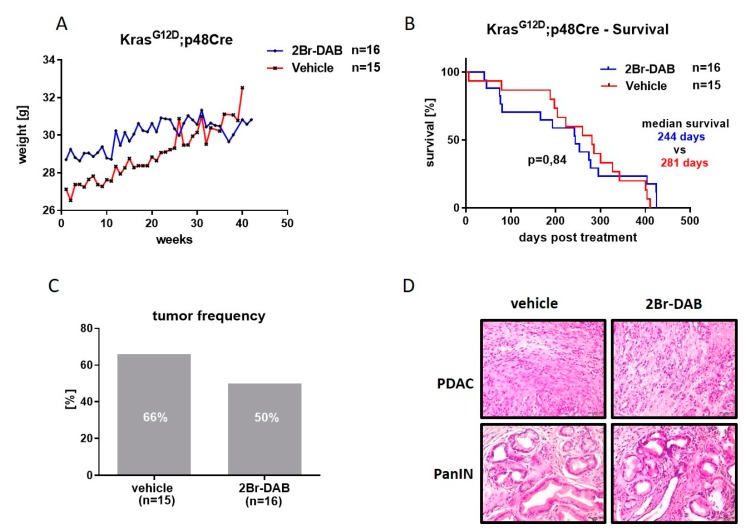
(**A**): Weight development in *LSL-Kras^G12D/+^;p48-1-Cre* (KC)-mice weekly treated by intraperitoneal injection with either 2Br-DAB at 7 mg/kg bodyweight (blue curve) or vehicle (red curve). Depicted are mean values of *n* = 16 2Br-DAB-treated and *n* = 15 vehicle-treated mice. (**B**): Survival analysis upon treatment start of 2Br-DAB (*n* = 16) and vehicle (*n* = 15)-treated KC-mice shows comparable survival times for KC-mice (244 days versus 281 days, *p* = 0.84, log-rank-test). (**C**): Tumor frequency determined for 2Br-DAB (*n* = 16; tumor frequency 50%) and vehicle (*n* = 15; tumor frequency 66%) treated KC-mice (*p* = 0.34, Chi square test). (**D**): H&E-staining for KC-mice showing histologic appearance of pancreatic ductal adenocarcinoma (PDAC) and pancreatic intraepithelial neoplasia (PanIN) lesions found in 2Br-DAB and vehicle-treated animals.

Subsequently, we assessed the potential of 2Br-DAB to slow ADM/PanIN-tumor progression in the KC mouse model. To this end, KC mice were enrolled at five months of age, a time point where PanIN and ADM lesions are present. Mice were treated with 2Br-DAB (*n* = 16) or vehicle (*n* = 15) for 12 weeks unless the endpoint criteria were met before (Figure S2). In line with our pilot study, no significant weight loss was recorded in KC mice upon 12 weeks treatment (Figure 2A). The median survival after treatment initiation between 2Br-DAB and vehicle cohorts was 244 days versus 281 days, respectively (log-rank test, *p* = 0.84, Figure 2B). Interestingly, upon necropsy, tumor frequency was reduced in the 2Br-DAB cohort compared with vehicle-treated mice (50% versus 66%, *p* = 0.34; Figure 2C,D). Taken together, these results show for the first time that 2Br-DAB does not exert any relevant side effects in KC mice compared with vehicle-treated mice. Interestingly, tumor frequency was reduced in 2Br-DAB-treated mice, indicating a potential tumor preventing effect of 2Br-DAB treatment.

### 3.3. FMD 2Br-DAB—Therapeutic Intervention Study in KPC Mice 

**Figure 3:**
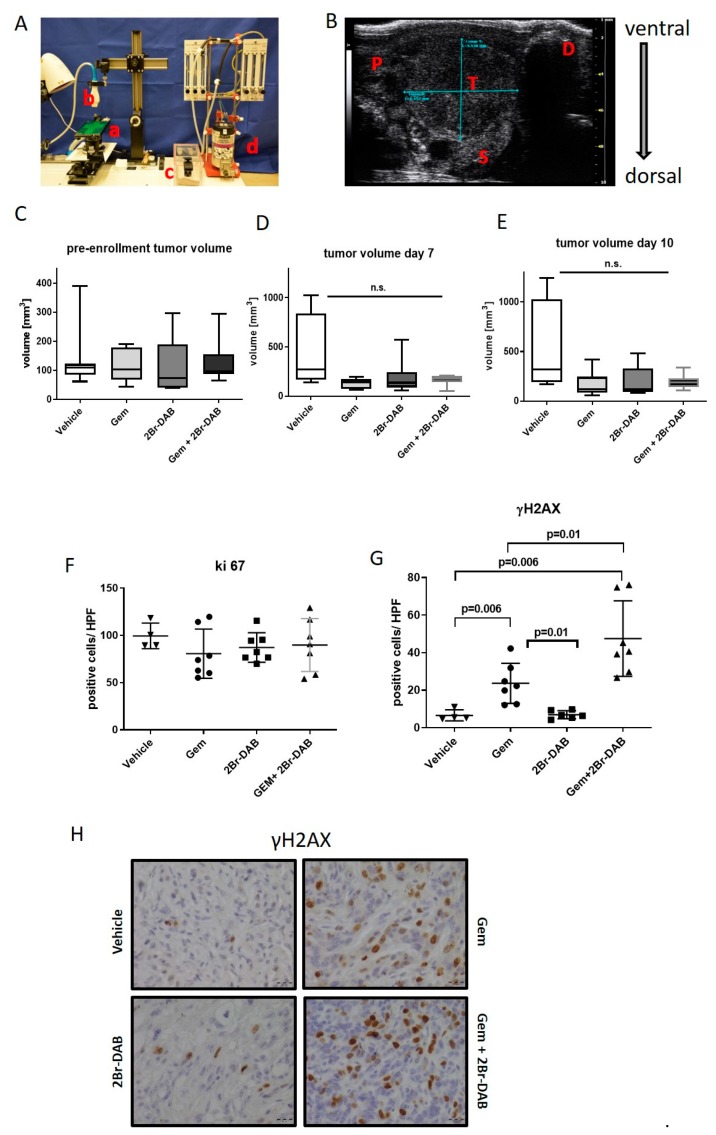
(**A**): High-resolution ultrasound system for tumor detection and volume quantification. a: working stage with anaesthetic nose cone. b: 4 MHz scan head. c: induction chamber. d: isoflurane vaporizer. (**B**): Anatomical structures during ultrasound examination. P: normal pancreatic tissue. D: duodenum with typical ultrasound extinction. S: part of spleen T: pancreatic tumor. (**C**): Absolute pre-enrollment tumor volumes obtained prior to treatment start for vehicle (*n* = 7), gemcitabine (*n* = 7), 2Br-DAB (*n* = 7), and gemcitabine + 2Br-DAB (*n* = 7). No significant difference between cohorts was seen. (**D**,**E**): Tumor volume increase at day 7 and day 10 by treatment cohorts. (**F**,**G**): Quantification of Ki67 and γH2AX immunohistochemistry of all treatment cohorts. For each mouse, at least seven high power fields (HPFs) were acquired at 40x magnification and manually quantified using ImageJ (Mann–Whitney U Test). (**H**): Representative images of γH2AX immunohistochemistry.

To investigate the anti-neoplastic effects of 2Br-DAB on established tumors, we employed the KPC mouse model for a therapeutic intervention study. KPC mice develop endogenous, desmoplastic pancreatic tumors from two months of age [28]. Therefore, we used manual palpation and high-resolution ultrasound based screening for the detection and evaluation of tumor volumes according to previously described protocols (Figure 3A,B) [29,37]. Thus, KPC mice with comparable tumor volumes were randomly enrolled into the following treatment cohorts: vehicle (*n* = 7), gemcitabine monotherapy (*n* = 7), 2Br-DAB (*n* = 7), and the combination of 2Br-DAB and gemcitabine (*n* = 7) (Figure S3, Figure 3C). High-resolution ultrasound was performed again at day 7 and day 10 to monitor tumor growth during therapy. At day 7 and 10 after therapy initiation, KPC tumors slowed tumor growth upon gemcitabine and 2Br-DAB monotherapy without showing a statistical benefit for 2Br-DAB over gemcitabine (Figure 3D,E). The combination of 2Br-DAB and gemcitabine reached the most robust results for impaired tumor growth (Figure 3D,E). However, as tumor volumes determined by high-resolution ultrasound may miss meaningful biological anti-tumoral effects of chemotherapeutic agents as a result of large amounts of cellular and non-cellular stromal components that are not altered or depleted during chemotherapy, we performed an immunohistochemical analysis of formalin-fixed paraffin embedded (FFPE) tumor tissue. While overall proliferation did not show significant differences among the treatment cohorts (Figure 3F), γH2AX as a marker for DNA-damage was significantly increased upon gemcitabine and combination therapy with 2Br-DAB and gemcitabine, whereas 2Br-DAB monotherapy did not show any significant effects compared with the vehicle cohort (Figure 3G,H). 

### 3.4. Combination of Radiation Therapy and 2Br-DAB In Vitro and In Vivo 

**Figure 4:**
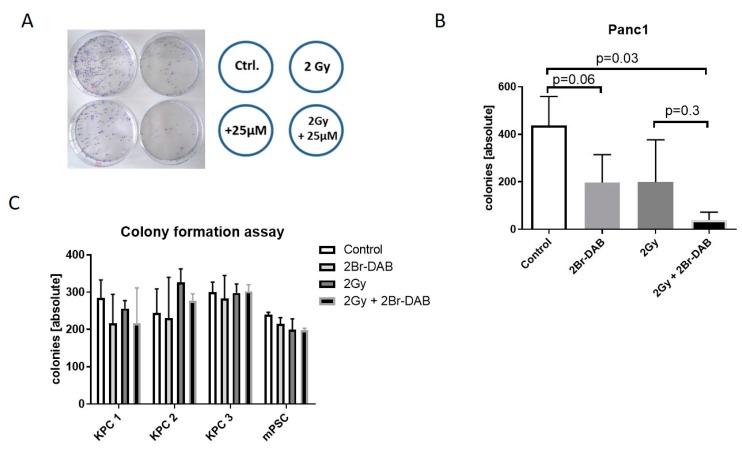
(**A**): Colony forming assay for pancreatic cancer cell lines. Panc1 cells were seeded in six-well plates (30,000 cells/µL), incubated with 25 µM DAB for 24 h, irradiated (2 Gy), and brought out in triplicates on 100 mm dishes (3000 cells/dish). Quantification was done manually 14 d later. (**B**): Quantification of manually counted colonies. For the Panc1 cell line, 2Br-DAB and irradiation led to a significant reduction of colony formation (results of two independent biologic experiments) (Mann–Whitney U test). (**C**): Colony-forming assay for murine pancreatic cancer cell lines (experimental design as mentioned in (**A**)).

Data from large phase III trials have shown no benefit of radiotherapy or chemoradiation over chemotherapy alone in PDAC [38,39,40,41]. To test whether 2Br-DAB acts as radiosensitizer in human and murine pancreatic cancer cell lines, we performed colony formation assays with 2Br-DAB treatment followed by irradiation [19,20]. After 24 h pre-incubation with low dose 25 µM 2Br-DAB, cells were exposed to 2 Gy X-ray irradiation. For Panc1 cells, 2Br-DAB treatment as well as 2 Gy irradiation robustly reduced colony formation by >50% of the controls (Figure 4A,B). A combination of both treatments further decreased colony formation, suggesting a modest radiosensitizing effect of 2Br-DAB (Figure 4A,B); *p* = 0.03). For BxPC3, no effect of radiation or chemoradiation with 2 Gy and low dose 2Br-DAB was observed on colony formation capacity, most likely because of the substantially higher GI_50_ for 2Br-DAB (Figure 1B). In contrast to human Panc1 cells, murine KPC cell lines 1–3 as well as murine pancreatic stellate cells (mPSCs) showed no relevant effect on colony formation upon treatment with low dose 2Br-DAB, 2 Gy irradiation, or the combination of both (Figure 4C). Subsequent dose escalation experiments in KPC 1–3 cell lines showed reduction of colony formation capacity upon increasing concentrations of 2Br-DAB; however, a radiosensitizing effect of 2Br-DAB (range: 2–8 Gy) was not observed (Figure S4). 

**Figure 5:**
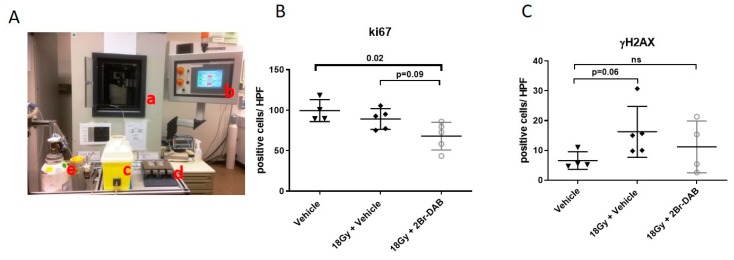

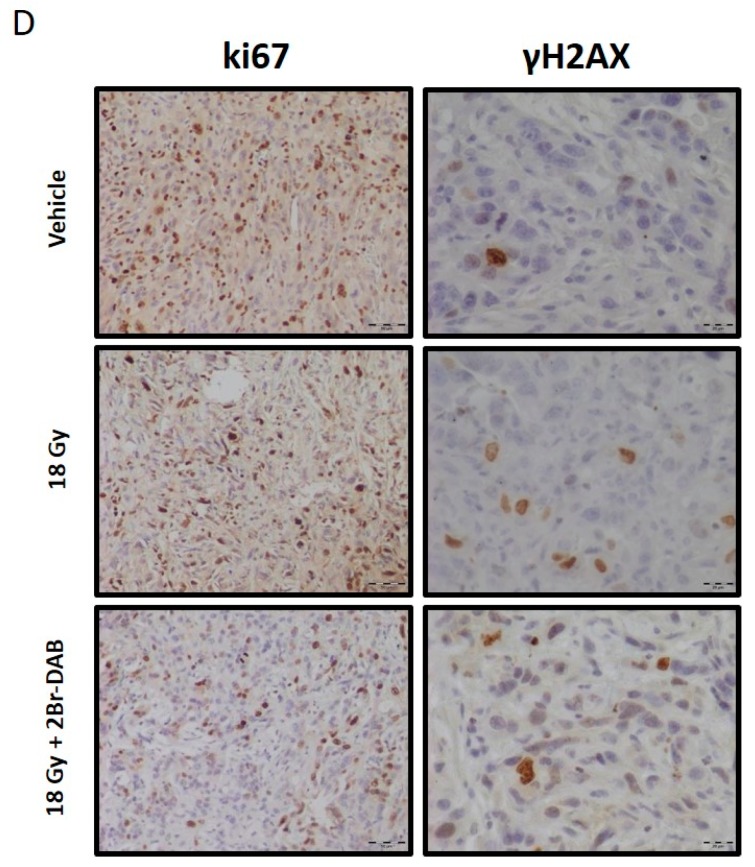
(**A**): Experimental set-up for radiotherapy of KPC mice. a: Irradiation chamber b: control table c: anesthetic induction chamber d: working stage including mouse fixation system and 25 mm thick lead plates. e: oxygen supply and isoflurane vaporizer. (**B**,**C**). Quantification of Ki67 and γH2AX immunohistochemistry. Ki67 positivity is significantly decreased upon combination of 2Br-DAB and 18 Gy (*p* = 0.02; Mann–Whitney U Test). (**D**): Representative images of γH2AX and Ki67 immunohistochemistry for all three treatment cohorts.

To investigate the effect of radiotherapy and 2Br-DAB in vivo, we enrolled tumor-bearing KPC mice with comparable tumor volumes in two separate cohorts and treated them for 12 days with either vehicle (*n* = 7), radiotherapy + vehicle (*n* = 6), or radiotherapy + 2Br-DAB (*n* = 5; Figure S5, Figure 5A). The radiation was applied 1 h post-injection of 2Br-DAB at 7 mg/kg. In total, a cumulative radiation dose of 18 Gy was applied to tumor-bearing mice. Tumor growth was monitored by high-resolution ultrasound at day 7 and day 10. The day 7 ultrasound showed no significant reduction of tumor volumes in the radiotherapy + 2Br-DAB cohort compared with vehicle mice (Table 1). After 10 days of treatment, the smallest pancreatic tumors were present in the combination cohort of radiotherapy and 2Br-DAB; however, no statistical significance was reached (Table 1). Interestingly, molecular analysis of FFPE tumor tissues showed a significantly reduced proliferation rate for the combination treatment (Figure 5B, *p* = 0.02), and increased numbers of γH2AX positive tumor cells in both treatment cohorts compared with vehicle mice, although without reaching statistical significance (Figure 5C). Thus, despite the lack of a clear radiosensitizing effect of 2Br-DAB in murine KPC tumor cells in vitro, combination therapy of 2Br-DAB and 18 Gy showed promising anti-tumor activity with smaller KCP tumors, a significantly reduced proliferation rate, and enhanced DNA damage.

## 4. Discussion

The pronounced resistance to standard chemo- and radiotherapy is a hallmark feature of PDAC. While the value of radiotherapy and chemoradiation is still controversially debated in PDAC, novel intensified chemotherapies have recently emerged for the treatment of PDAC. To this end, FOLFIRINOX and gemcitabine + nab-paclitaxel are two regimens for stage IV PDAC patients that have been shown to prolong survival [5,6]. However, these regimens can only be offered to patients with good performance status as the improved effectiveness is traded against an increased frequency of severe side effects. Consequently, there is still a considerable number of patients with metastatic PDAC that will only receive gemcitabine monotherapy or best supportive care with a median survival of 6–8 months [7]. Therefore, novel targeted therapies are urgently needed for the treatment of early, locally advanced, and metastatic PDAC to improve the devastating prognosis of patients. 

Here, we investigated the therapeutic potential of a novel non-platinum based halogenated compound (an FMD compound), 2Br-DAB, in pancreatic cancer for the first time. 2Br-DAB is structurally related to cisplatin. Previous studies have investigated a potentially selective cytotoxic mechanism of FMDs to target neoplastic cells with little toxicity towards normal cells or tissues [19,20]. The specificity against cancer cells was explained by the presence of a reductive environment in neoplastic cells that facilitates the DET reaction and causes DNA single- and double-strand breaks. 

Our in vitro results suggest robust anti-proliferative effects of 2Br-DAB in a broad range of human and murine pancreatic cancer cell lines. However, preclinical results from cell lines or xenograft models could often not sufficiently be translated to human PDAC in the past [42]. One prevailing hypothesis is that the pronounced tumor microenvironment that is found in human PDAC is not recapitulated in 2D cell lines and subcutaneously transplanted tumor models [30,32,37,43,44,45]. To this end, the introduction of GEMMs has marked a milestone in pancreatic cancer research, and in particular, the KC and the KPC models provide excellent preclinical platforms for testing novel therapeutic compounds.

Notably, 2Br-DAB was extremely well tolerated in vivo with no observed side effects even after long term treatment over 12 weeks, consistent with previous studies [19,20]. Therefore, we tested the potential of 2Br-DAB to slow PanIN-tumor progression in the KC model. Although we did not detect significant differences in overall survival, the decreased rate of invasive carcinomas upon long-term administration of 2Br-DAB may indicate a potential chemopreventive effect that should be further explored, preferable in high-risk individuals such as patients with BRCA mutations, Peutz–Jeghers syndrome, or chronic pancreatitis [46].

In order to test the potential antineoplastic effects of 2Br-DAB, we employed the highly aggressive and chemoresistant KPC model. Upon enrollment, all mice had developed highly proliferative and invasive pancreatic tumors that closely resemble human PDAC at advanced stages including a dense stromal reaction. In analogy to humans, gemcitabine is only mildly efficacious in KPC mice, and similar effects could be observed for 2Br-DAB. Tumor volumes initially decreased compared with vehicle treatment, but gradually increased again after prolonged treatment. However, molecular analysis suggested that the combination of 2Br-DAB and gemcitabine might have synergistic effects, as evidenced by the elevated numbers of γH2AX positive cells.

The conflicting results regarding the radiosensitizing effect of 2Br-DAB in vitro for human and mouse pancreatic cancer cell lines might be the result of inter-species differences, or the fact that the radiosensitizing effect is most prominent at low doses of 2Br-DAB in Panc1 cells. However, we attempted to investigate the combination of radiotherapy and 2Br-DAB in a first pilot study using the KPC mouse model. To the best of our knowledge, radiotherapy has not been evaluated in the KPC mouse model before. Here, we provide first data in the KPC model showing that radiotherapy is a feasible experimental approach to treat endogenous pancreatic tumors. To reduce off-target effects of the radiation, in particular to abdominal organs such as small and large bowel, KPC tumors were located by high-resolution ultrasound prior to radiation. Interestingly, tumors that received radiation and 2Br-DAB were smaller on ultrasound and showed significantly reduced proliferation rates at endpoint. Potentially, this effect could be further increased if the cumulative radiation dose of 18 Gy would be elevated. However, we could not assess overall survival in KPC mice as the treatment schedule with irradiations, ultrasound examinations (including repeated sedations), and drug administration is not feasible for longer than two weeks. Magnetic-resonance imaging-guided imaging approaches may be suitable to further optimize targeted radiotherapy in mice [47].

In summary, 2Br-DAB reduces the viability of murine and human pancreatic cancer cells in a dose-dependent manner in vitro. Furthermore, 2Br-DAB exerts modest chemopreventive and anti-tumoral activity in the KC and KPC models, and can be safely combined with gemcitabine or radiotherapy. Thus, our study provides first experimental data on 2Br-DAB and radiotherapy in relevant mouse models of PDAC. 

## Figures and Tables

**Table 1:** Mean ultrasound volumes of *LSL-Kras^G12D/+^;LSL-Trp53^R172H/+^;Pdx-1-Cre* (KPC) tumors determined at enrolment, and at day 7 and day 10 of the respective treatment.

Cohort.	Pre-Enrollment (mm^3^)	7 Days (mm^3^)	10 Days (mm^3^)
Vehicle (*n* = 7)	138.2	425.3	404.1
18 Gy + Vehicle (*n* = 6)	188.7	343.8	373.4
18 Gy + 2Br-DAB (*n* = 5)	170.6	210.3	218.1

## Supplementary Materials

The following are available online at https://www.mdpi.com/2073-4409/8/6/563/s1.
