# Supplementary material for: Preclinical Evaluation of 1,2-Diamino-4,5-Dibromobenzene in Genetically Engineered Mouse Models of Pancreatic Cancer
Source: Cells. 2019 Jun 9;8(6):563. doi: 10.3390/cells8060563 (PMC6627568; doi:10.3390/cells8060563)
Supplement: Supplementary file 1 [file cells-08-00563-s001.pdf]

Figure S1:

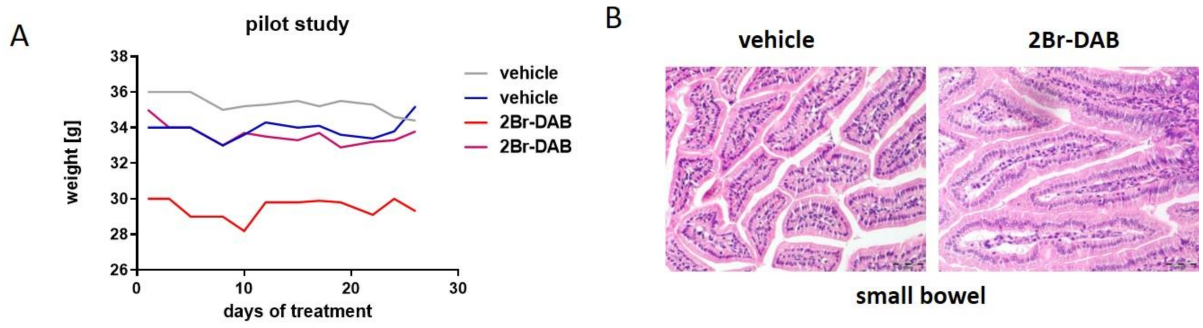

Figure S1: A: Weight curve of KC-mice treated with either 2Br-DAB (n=2) or vehicle (n=2) three times per week for 30 days. **B:** Representative small bowel H&E-staining showed no gastrointestinal toxicity.

Figure S2:

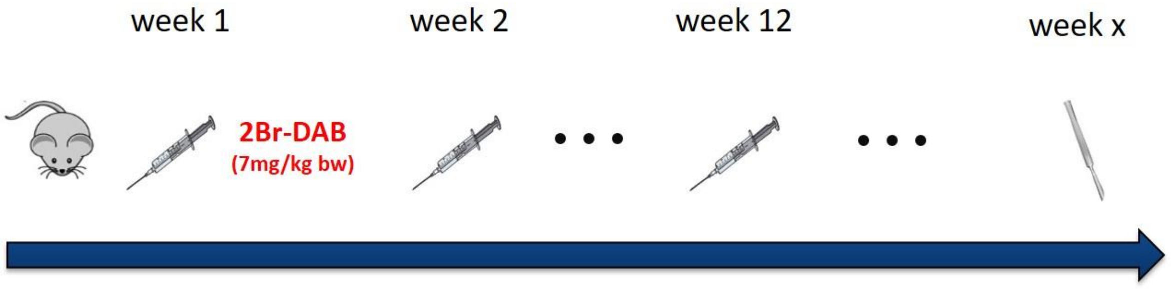

Figure S2: Treatment schedule for KC-mice. Five months old KC-mice were randomly distributed to either weekly intraperitoneal 2Br-DAB or vehicle intraperitoneal injection. After 12 weeks mice were weekly checked until endpoint criteria were reached.

Figure S3:

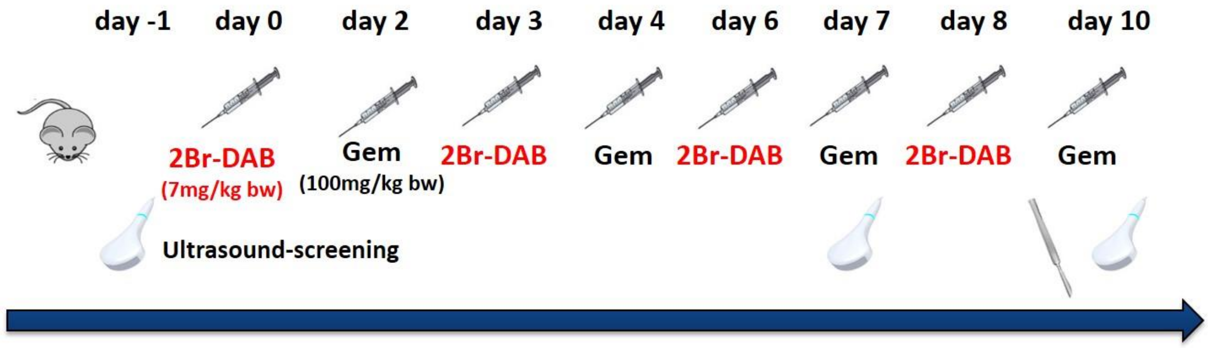

Figure S3: Treatment schedule for intervention study. Mice were injected with vehicle, 2Br-DAB, gemcitabine or a combination of gemcitabine + 2Br-DAB. Ultrasound was performed at day -1, -7 and 10 to calculate tumor volumes.

Figure S4

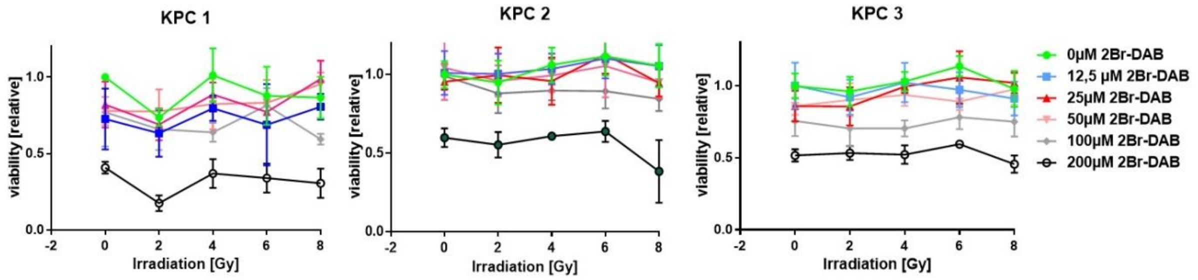

Figure S4: *In vitro* radiotoxicity assay of 2Br-DAB. Cell viabilities of murine PDAC cell lines (KPC1-3) after a 60min pre-treatment of 2Br-DAB with various concentrations at 0–200µM followed by 2Gy-8Gy irradiation. 72hours post-irradiation cell viability was measured by MTT assay.

Figure S5

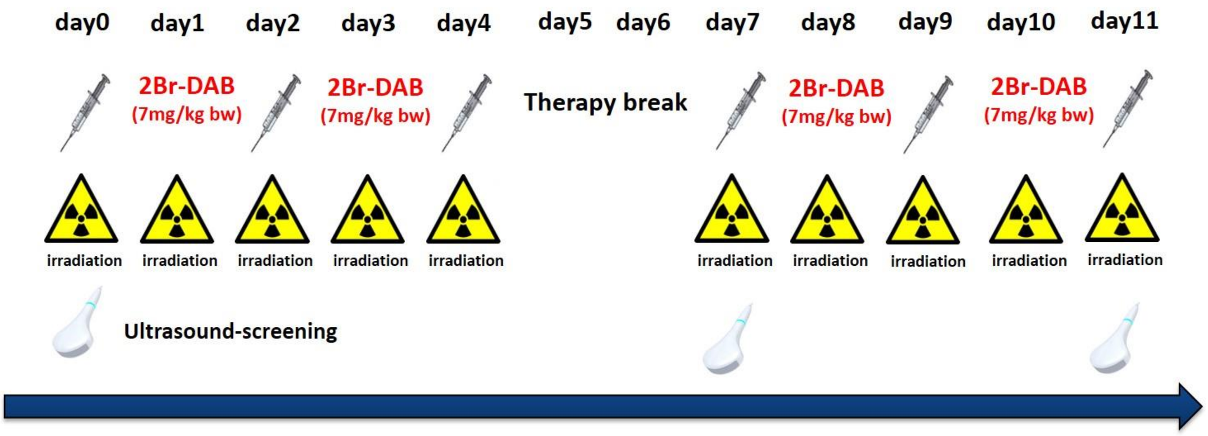

Figure S5: Treatment schedule for the KPC irradiation study. Irradiation was applied 1h after 2Br-DAB administration.
